# Red Cell Distribution Width and Inappropriateness of Left Ventricular Mass in Patients with Untreated Essential Hypertension

**DOI:** 10.1371/journal.pone.0120300

**Published:** 2015-03-20

**Authors:** Lizhang Chen, Zhanzhan Li, Yanyan Li, Jing Xue, Peng Chen, Shipeng Yan, Caixiao Jiang, Yingyun Hu, Xing Qiao

**Affiliations:** 1 Department of Epidemiology and Health Statistics, School of Public Health, Central South University, Changsha, Hunan Province, China; 2 Department of emergency, Henan Provincial Hospital of Traditional Chinese Medicine, Zhengzhou, Henan Province, China; 3 Xiangya Medical School, Central South University, Changsha, Hunan Province, China; 4 Hunan provincial tumor hospital, Changsha, Hunan Province, China; 5 Department of Plastic Surgery, First Teaching Hospital of Xinjiang Medical University, Urumqi, Xinjiang Uygur Autonomous Region, China; University of Buenos Aires, Faculty of Medicine, Cardiovascular Pathophysiology Institute, ARGENTINA

## Abstract

**Background:**

Left ventricular hypertrophy (LVH) was suggested to be an important risk factor for hypertensive vascular complications. Previous studies had also shown that red cell distribution width (RDW) was associated with morbidity and mortality of cardiovascular disease. However, few have yet investigated possible association between RDW and LVH. The aim of the present study was to evaluate the relationship between LVH and RDW levels in hypertensive patients.

**Methods:**

Physical examination, laboratory tests and echocardiography were conducted in 330 untreated newly diagnosed hypertensive patients attending the cardiology consultation unit at the Anzhen Hospital of Beijing. The multivariate logistic regression model was used to verify the independent association between RDW and LVH.

**Results:**

174 patients without LVH and 156 patients with LVH were rolled in the study. The patients with LVH had higher mean SBP, albumin to creatinine ratio, total cholesterol, RDW and fasting glucose compared with non-LVH group. The mean HDL-cholesterol level was significantly lower in patients with LVH than patients without LVH. The multiple logistic regression model suggested that patients with a higher RDW level were more likely to be LVH (OR=2.187, 95%CI: 1.447-3.307, *P*<0.001). Other predictive factors for LVH were mean SBP, serum creatinine, glucose level. The receiver operating characteristics (ROC) curves indicated area under the curve was 0.688(95%CI: 0.635-0.737, P<0.001) with a cut-off value of 12.9, the RDW predicted LVH status among hypertensive patients with a sensitivity of 72.4% and a specificity of 60.3%.

**Conclusions:**

The higher RDW level was observed in the LVH group compared with the non-LVH group. RDW might be associated with LVH in hypertensive patients. These data highlight the role of RDW as a predictor of organ damage in essential hypertensive patients.

## Introduction

According to 2013 European Society of Hypertension/Cardiology guidelines, the presence of subclinical organ damage is a fundamental and important factor in determining the estimated cardiovascular risk with proposed scale [1]. Left ventricular hypertrophy (LVH) is one of common subclinical organ damage induced by hypertension, and the presence of LVH increases mortality of cardiovascular disease [2–4]. According to some statistics, 20–40% of hypertensive patients have the complication of LVH [5]. Identifying specific risk factor index for hypertensive patients has therefore great potential for preventing adverse cardiovascular events.

As an index of the routine blood cell count, red cell distribution width (RDW) is not only used to evaluate different types of anemia but also is a potent predictor of morbidity and mortality in a variety of settings, especially in many cardiovascular diseases. Several lines of evidences had suggested increased RDW was associated with a higher possibility of varieties of diseases. It is observed that increased RDW level appears to be an independent predictor of coronary heart disease risk in the general population [6]. Sánchez-Chaparro et al. conducted a cross-sectional study with 217,567 Spanish working people undergoing a routine physical checkup, and found that higher RDW level was associated with metabolic syndrome (MetS), a well-known multiple risk factors for cardiovascular disease [7]. The recent study also demonstrated that RDW is a useful marker in predicting inadequate reduction in nighttime BP in essential hypertension, and RDW values are closely related with inflammation status in hypertensive patients [8]. Hypertension is an inflammatory disorder. Previous studies have indicated a significant association between hypertension and inflammatory process [9, 10]. The correlation between RDW and hypertension has been explored in a number of studies [11, 12].

Inflammatory status is significantly related to ineffective erythropoiesis, and it has been suggested that inflammatory cytokines such as interleukin (IL)-1 β, IL-6, tumor necrosis factor (TNF)-α, desensitize bone marrow elytroid progenitors to erythropoiesis, inhibit red blood cell maturation and thereby promote anysocytosis [13, 14]. Considering the above situation, we suppose that high blood pressure does damage to endotheliocyte promoting the secretin of inflammatory cytokines, and some inflammatory cytokines such as vascular endothelial growth factor (VEGF) play an important role in inducing myocardial hypertrophy [15]. Therefore, as a sensitive index of inflammatory status in this process, RDW could be a potential predictor of LVH in essential hypertensive patients. Various hematological and inflammatory biomarkers, such as mean platelet volume (MPV) [16], IL-10, IL-6 [17] have been used in clinical practice to evaluate related target organ damage in hypertensive patients. However, few have yet investigated possible association between RDW and LVH. The aim of the present study was to evaluate the relationship between LVH and RDW levels in hypertensive patients.

## Materials and Methods

### Study population

In a cross-sectional study, data from 330 patients visiting the Cardiology Center at Anzhen Hospital of Beijing from November 2012 to October 2013 were analyzed. Hypertension was defined as systolic BP (SBP)≥140 mmHg or a diastolic BP (DBP)≧90 mmHg and not treated with anti-hypertensive drug [18]. Among 513 patients who underwent physical examination, laboratory tests and echocardiography for evaluation of cardiac function, 330 patients’ data (S1 Dataset) were collected by applying an exclusion criterion. According to echocardiographic examination, 156 patients with left ventricular hypertrophy (LVH) and 174 patients without LVH were enrolled in the present study. The patients’ clinical and demographic characteristics including age, sex, and smoking habits were obtained through a standardized questionnaire. This study protocol was approved by the institutional review board of Xiangya Medical School, and written informed consent was obtained from all participants.

Exclusion criteria were as follows: patients who ever received treatment for hypertension; patients with secondary hypertension, heart failure, diabetes mellitus, renal or hepatic dysfunction, clinical evidence of cancer, systemic inflammatory disease, hematological system disorder and known coronary artery or cerebrovascular disease; disabling diseases such as dementia; and inability to cooperate.

### Laboratory examination

Hematologic testing was performed on the ADVIA 120 (Bayer Diagnostics, Newbury, Berkshire, UK) automated hematology analyzer, which measures hemoglobin photometrically, including white blood cell counts, platelet counts, hemoglobin, mean corpuscular volume and RDW, optical laser light scattering for cell enumeration, flow cytometer and laser diffraction for red blood cell (RBC) counts. Serum creatinine (Scr) was measured on a Roche/Hitachi Modular System P (Roche Diagnostics GmbH, Mannheim, Germany) by creatinine Jaffe´, rate blanked and compensated assay. Microalbuminuria was defined as that urine albumin-creatinine ratio (ACR) is between 30 μg /mg and 300 μg/mg [19]. Urine albumin concentration was measured by immunoturbidimetric method [20]. In addition, fasting blood glucose level, creatinine level and fasting serum lipid status including total cholesterol, low-density lipoprotein (LDL), high-density lipoprotein (HDL), triglyceride, and high-sensitive C-reactive protein levels were also recorded. Body mass index (BMI) was calculated as weight (kg) divided by height squared (m^2^).

### Echocardiography

All echocardiographic studies were performed on each subject by a single sonographer with a commercially available machine (Achieva, Philips Medical Systems) using a 1–5 MHz transducer. The overall single dimensional left ventricular measurements and the 2-dimension views were obtained according to the standard recommended by American Society of Echocardiography [21, 22]. To reduce bias, the cardiologist was blinded to the results of urinalysis. Left ventricular mass was calculated by the following formula, LVM [23] = 1.04*0.8*{(VST_d_ * LVID_d_ * PWT_d_) ^3^-(LVID_d_) ^3^} + 0.6, where IVS_d_ is diastolic interventricular septum, LVD_d_ is diastolic left ventricular dimension, and PWT_d_ is diastolic posterior wall thickness. LVMI = LVM/BSA (Body Surface Area, BSA). LVH was defined as following: LVMI ≥ 125 g/m^2^ in men and ≥ 110 g/m^2^ in women [24].

### Statistical analysis

Data were reported as mean ± standard deviation for quantitative variables and as percentages for qualitative variables. RDW was examined as a continuous variable. Differences between quantitative variables were tested by Student t test. Differences between qualitative variables were tested using Chi-squared test. Pearson’s correlation coefficients were calculated if appropriate. Multiple logistic regression analysis was used to calculate the odds ratio of independent variables. The receiver operating characteristics (ROC) curves explored the relationship between RDW and LVH. Statistical analysis was performed using SPSS version 17.0 (SPSS Inc, USA), GraphPad Prism 5 and MedCalc (http://www.medcalc.org/), and the level of statistical significance was defined as *P*<0.05.

## Results

### General characteristics of the subjects

**Fig 1:**
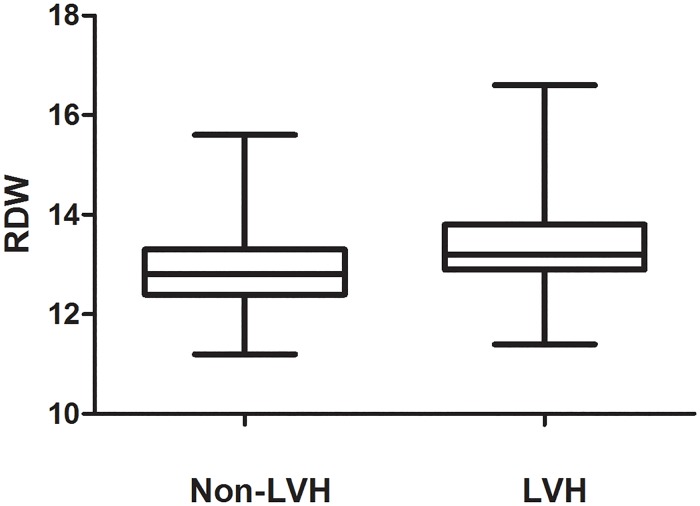
Comparison of red cell distribution width (RDW) level between the left ventricular hypertrophy (LVH) group and non-LVH group.

As shown in Table 1, 174 patients without LVH and 156 patients with LVH were enrolled in the study. Among the 330 hypertensive patients, 19 were diabetes mellitus. In the comparisons of demographic, clinical, and echocardiographic characteristic between hypertensive patients with LVH and without LVH, the average age was 46.8±7.9 years and 46.3±7.7 years respectively. The sex ratio (female/male) in the LVH group is 0.54 and 0.79 in the non-LVH. The patients with LVH had higher mean SBP (138.8±12.3 vs133.4±9.3, *P*<0.001), albumin to creatinine ratio (47.9±56.4 vs 32.1±39.2, *P* = 0.003), total cholesterol (5.4±0.8 vs 5.2±0.9, *P* = 0.011), RDW (13.3±0.6 vs 12.8±0.8, *P*<0.001, Fig. 1) and fasting glucose (5.8±0.6 vs 5.6±0.6, *P* = 0.021) compared with non-LVH group. The mean HDL-cholesterol level was significantly lower in patients with LVH than patients without LVH (1.1±0.2 vs 1.2±0.2, *P*<0.001).

**Table 1:** Clinical characteristics of hypertensive patients with and without LVH.

	left ventricular hypertrophy		
Parameters	No(n = 174)	Yes(n = 156)	*P*
Demographic			
Age, y	46.8±7.9	46.3±7.7	0.503
Sex			0.096
Male	77(58.3%)	55(41.7%)	-
Female	97(49.0%)	101(51.0%)	-
Smoking, yes	22(12.6%)	27(17.3%)	0.234
Diabetes mellitus, yes	7(4.0%)	12(7.7%)	0.153
Body mass index, kg/m^2^	26.0±3.5	26.1±3.6	0.626
Clinical index			
Mean SBP*, mmHg	133.4±9.3	138.8±12.3	<0.001
Mean DBP*, mmHg	87.0±7.9	88.8±10.4	0.083
Triglyceride, mmol/dL L	1.8±0.5	1.9±0.5	0.392
HDL-cholesterol, mmol/dL	1.3±0.2	1.1±0.2	0.024
LDL- cholesterol, mmol/dL	3.2±0.9	3.1±0.8	0.129
Total cholesterol, mmol/dL	5.2±0.9	5.4±0.9	0.010
Fasting glucose, mmol/dL	5.6±0.6	5.8±0.6	0.021
Albumin to creatinine ratio, mg/g	32.1±39.2	47.9±56.4	0.003
Serum creatine, mmol/dL	67.3±13.0	71.1±13.2	0.008
Uric acid,	341.9±50.0	347.±47.4	0.280
Red blood cell, ×10^12^/L	4.9±0.4	4.9±0.4	0.992
Red cell distribution width (%)	12.8±0.6	13.3±0.8	<0.001
Hs-CRP*, mg/dL	2.3±5.4	2.6±4.5	0.678
Hemoglobin,g/L	148.5±14.7	149.2±15.5	0.711
Echocardiographic examination			
IVST*, mm	9.0±1.0	10.5±1.2	<0.001
LVEDd*, mm	45.4±4.5	49.5±4.0	<0.001
LVESd*, mm	28.5±4.0	31.1±3.5	<0.001
PWT*, mm	9.0±1.9	10.3±1.3	<0.001
Ejection fraction, %	66.8±5.3	64.7±7.1	0.002
Left ventricular mass, g	169.3±30.5	236.7±43.9	<0.001
Left ventricular mass index, g/m2	93.5±12.2	129.7±19.4	<0.001

*SBP: systolic blood pressure; DBP: diastolic blood pressure; Hs-CRP: high-sensitive C-reactive protein, IVST: interventricular septal thickness, LVEDd: left ventricular end-diastolic diameter, LVESd: left ventricular end-systolic diameter, PWT: posterior wall thickness

The results of echocardiographic examination shown that patients with LVH had significantly longer interventricular septal thickness (IVST: 10.5±1.2 vs 9.0±1.0, *P*<0.001), left ventricular end-diastolic diameter (LVEDd: 49.5±4.0 vs 45.4±4.5, *P*<0.001), left ventricular end-systolic diameter (LVESd: 31.1±3.5 vs 28.5±4.0, *P*<0.001), posterior wall thickness (PWT: 10.3±1.3 vs 9.0±1.9, *P*<0.001). The LVH group had significantly lower ejection fraction than the non-LVH group (EF, 64.7±7.1 vs 66.8±5.3, *P* = 0.002). The mean left ventricular mass for two groups was 236.7±43.9 and 169.3±30.5 (*P*<0.001). Among these echocardiographic indexes, LVM, LVMI, EF, and PWT were correlated with RDW in hypertensive patients. LVM (r = 0.24, *P*<0.001), LVMI (r = 0.26, *P*<0.001), and IVST (r = 0.191, *P*<0.001) have a positive relationship with RDW while EF (r = -0.13, *P* = 0.016) suggested an inverse relationship with RDW (Table 2).

**Table 2:** Correlation coefficients of relation between red distribution width and some parameters.

	Correlation coefficients	P
Age	-0.023	0.083
Mean SBP	0.471	<0.001
Mean DBP	0.335	<0.001
left ventricular mass	0.241	<0.001
left ventricular mass index	0.264	<0.001
Interventricular septum thickness	0.095	0.085
Left ventricular posterior wall thickness	0.191	<0.001
Platelet	0.108	0.101
Ejection fraction	-0.132	0.016

**Fig 2:**
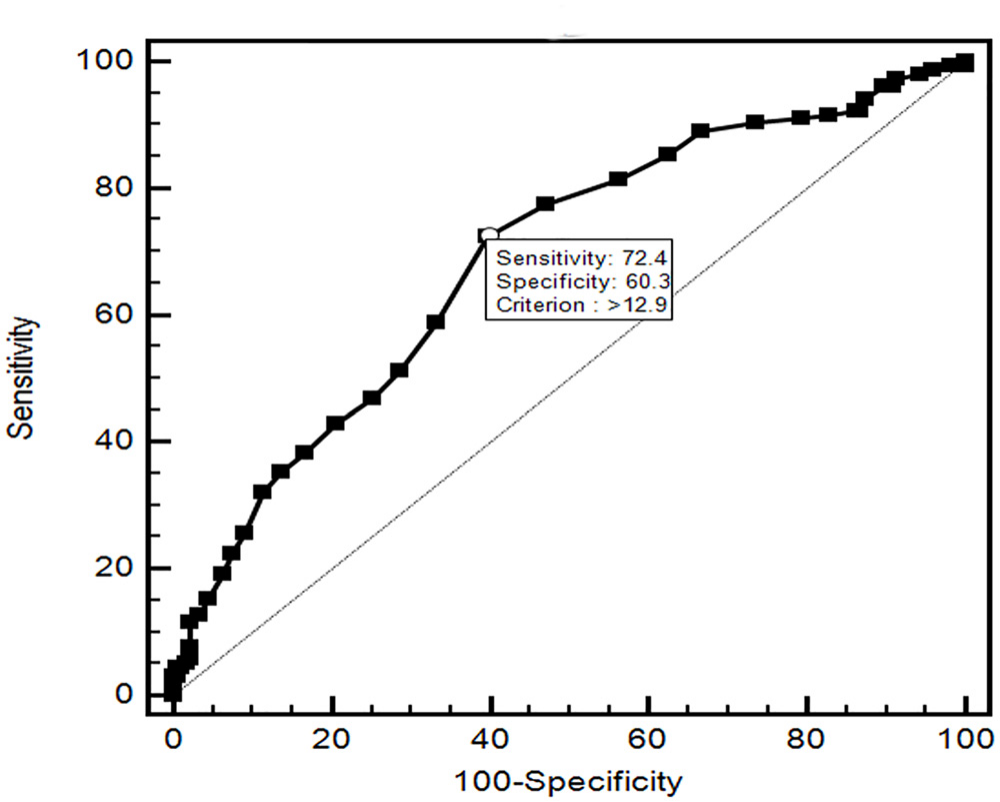
Receiver operating characteristic curve of red cell distribution width (RDW) for predicting left ventricular hypertrophy (LVH) in hypertensive patients.

A multivariable logistic regression model was built to assess the relationship between LVH and RDW, controlled for sex and smoking status as categorical variables and age, fasting glucose, serum creatinine, total serum cholesterol, SBP, albumin to creatinine ratio, total cholesterol and HDL-C as continuous independent variables. The results suggested that patients with a higher RDW were more likely to be LVH (OR = 1.829, 95%CI: 1.242–2.694, *P* = 0.002). Other predictive factors for LVH were SBP, serum creatinine, glucose level (Table 3). In hypertensive patients, the receiver operating characteristics (ROC) curves explored the relationship between LVH status and RDW. The area under the curve was 0.688 (95%CI: 0.635–0.737, *P*<0.001) with a cut-off value of 12.9, the RDW predicted LVH status among hypertensive patients with a sensitivity of 72.4% and a specificity of 60.3% (Fig. 2).

**Table 3:** Multivariable logistic regression analysis of prediction of LVH.

Variable	Odds ratio	95% confidence interval	*P*
Sex(Female)	0.589	0.279 to 1.245	0.166
Smoking, yes	0.672	0.306 to 1.478	0.323
Ag, y	0.985	0.954 to 1.018	0.373
Fasting glucose, mmol/dL	1.594	1.059 to 2.401	0.025
Total cholesterol, mmol/dL	1.345	1.009 to 1.794	0.043
HDL- cholesterol, mmol/dL	0.574	0.175 to 1.884	0.360
Mean SBP, mmHg	1.031	1.004 to1.058	0.021
Serum creatinine, mmol/dL	1.036	1.008 to 1.065	0.012
Albumin to creatinine ratio, mg/g	0.996	0.990 to 1.003	0.280
Red cell distribution width	2.187	1.447 to 3.307	<0.001

## Discussion

This study demonstrates the relationship between RDW and LVH in hypertensive patients. The present data indicates that RDW is significantly increased in hypertensive patients with LVH compared with non-LVH group. RDW >12.9 measured in patients who have been recently diagnosed as hypertension and who are not receiving any drug treatment had a 72.4% sensitivity and 60.3% specificity in predicting LVH. This result suggests cardiac compensation’s establishment in the early stages of hypertension and highlights the usefulness of RDW for the early detection of inflammatory status and as a potential marker of target organ damage in hypertensive patients. This study also indicates an association between RDW and LVH and affirms the importance of this index in the full evaluation of hypertensive patients. We noticed that a similar study conducted by Kilicaslan et al had been published in the Hypertension Research [25]. In this study four different geometric patterns (Normal geometry, concentric remodeling, eccentric hypertrophy, concentric hypertrophy) were determined according the LVMI, and they found that LV geometry type were independent associated with high RDW. But the present study encompasses a larger sample size (n = 330), which is more than doubled to theirs (n = 139). The operating characteristic curve analysis in their study only included concentric hypertrophy (n = 35), which makes the results statistically weak. It has been suggested that RDW is an inflammatory marker of CV diseases, and high-sensitive C-reactive protein, an important inflammatory marker is not assessed in their study. Besides, the result is also questionable in Baris’s study, the abstract part showed that LV geometry type is independently predictors of high RDW level, beta = 0.228, *P* = 0.01. However, the article part give such a wonder result: beta = 0.408, *P*<0.001.

The likely explanation of the association between RDW and LVH might be attributed to the presence of chronic inflammation. Chronic inflammation could cause RDW level elevation and increased RDW reflects an underlying chronic inflammation, which could result in a higher risk of cardiovascular disease [26]. Uysal et al. investigated the relationship between RDW and acute ST-elevated myocardial infarction (STEMI) in young patients and found young patients with STEMI had higher RDW level compared with young patients with normal coronaries [27]. Lappe et al. found that RDW was associated with mortality (correlating with hs-CRP levels) in patients with coronary artery disease [28]. But the CRP was not associated with LVH in our study. The reason could be that our results were obtained in a selected population without severe renal function impairment and free of cardiovascular diseases. The association between CRP and LVH would be more significant with the progress of hypertension. This also may highlights that RDW could be a more sensitive indicator in predicting LVH than CRP from the other side. Semba et al. investigated whether serum antioxidants and inflammation predicted RDW values in older women, and the patients in higher quartile of RDW were more likely to have a higher interleukin-6 level [29]. Animal model experiment found that the level of IL-6 in serum was lowered significantly after the Olmesartan Medoxomil completely reversed LVH of rats in renovascular hypertensive prevalence [17]. A previous study suggested IL-6 level was increased in hypertensive patients despite good blood pressure control, and IL-6 could cause cardiac hypertrophy through the IL-6 signal transducing receptor competent-glycoprotein 130[30, 31]. Inflammation may cause changes in red blood cell maturation by disturbing the red cell membrane, leading to increased RDW. Among these echocardiographic indexes, LVM, LVMI, EF, and PWT were correlated with RDW in hypertensive patients. LVM(r = 0.24, P<0.001), LVMI(r = 0.26, P<0.001), and IVST(r = 0.191, P<0.001) had a positive relationship with RDW level while EF (r = -0.13, P = 0.016) suggested an inverse relationship with RDW level.

Another important determinant of LVH in hypertensive patients might be oxidative stress that causes oxidative damage. The fact that increased oxidative stress led to activation of signaling pathways and induced cardiac hypertrophy have been found to prevent the development of cardiac hypertrophy [32, 33]. Cave et al. found that Cardiac hypertrophy induced by angiotensin II (Ang II), endothelin-1 (ET-1), tumour necrosis factor (TNF-α) or pulsatile mechanical stretch, in cultured cardiomyocytes has shown to be involved in intracellular reactive oxygen species (ROS) production, which was inhibited by antioxidants [34]. Takimoto et al. found that uncoupling the nitric oxide synthase-induced ROS production promoted the development of left ventricular hypertrophy during chronic pressure overload [35]. Semba et al. measured RDW at baseline, 12 months and 24 months in 786 moderately to severely disabled women aged more than 65 years old and found serum selenium was a potential predictor of RDW, which suggested that oxidative stress may be a potential underlying biological mechanism for increased RDW [36]. Generally, increased oxidative stress leads to the oxidation and damage of macromolecules, membranes, DNA and enzymes involved in cellular function and homeostasis [37]. Therefore, activation of key mediators of metabolic regulation by ROS as well as depletion or decreased activity of endogenous antioxidants promotes the pathological development of RDW [38].

The present study had several limitations that should be addressed. Frist, because it is limited to a cross-sectional design, establishing causal relationship between RDW and LVH is not sufficient. Second, the study population only included these hypertensive patients without receiving drug treatment. It needs to be further confirmed in the future study. Third, there are some points that RDW alone without other inflammatory markers may not give information to clinicians about the endothelial inflammatory condition of patients [39], and RDW should be accessed together with other serum inflammatory markers. But in the multiple regression, controlled for sex and smoking status as categorical variables and age, fasting glucose, serum creatinine, total serum cholesterol, SBP, albumin to creatinine ratio, total cholesterol and HDLC as continuous independent variables, and the analysis still gave a significant result (OR = 2.187, 95%CI: 1.447–3.307, *P*<0.001). Finally, although we demonstrated a significant association between elevated RDW and LVH, we could not determine the exact mechanism of this association.

In conclusion, the present study suggested RDW is a useful tool in predicting LVH in hypertensive patients without treatment. The higher RDW level was observed in the LVH group compared with the non-LVH group. RDW might be associated with LVH in hypertensive patients. These data highlight the role of RDW as a predictor of organ damage in essential hypertensive patients.

## Supporting Information

S1 Dataset Original data file. (XLS)
